# Diagnostic accuracy of optical coherence tomography angiography for choroidal neovascularization: a systematic review and meta-analysis

**DOI:** 10.1186/s12886-019-1163-5

**Published:** 2019-07-26

**Authors:** Rui Wang, Zhenzhen Liang, Xin Liu

**Affiliations:** 1 Epidemiology and Statistics, School of Public Health, Jilin University, Changchun, 130021 China; 2 Department of NHC Key Laboratory of Radiation Biology, Jilin University, Changchun, 130021 China

**Keywords:** Optical coherence tomography angiography, Choroidal neovascularization, Diagnosis, Meta-analysis

## Abstract

**Background:**

Optical coherence tomography angiography (OCTA), an innovative image technique, renders visualization of ocular neovascularization through non-invasive means, which has been applied in recent years. Therefore, the present study was designed to assess the diagnostic value of OCTA in detecting the choroidal neovascularization (CNV).

**Methods:**

In brief, PubMed, Web of Science and Cochrane Library databases were systematically searched from January 2014 to June 2019. Afterwards, a meta-analysis was performed to determine the pooled diagnostic accuracy in a random-effects model using STATA 15.1 and Meta-Disc 1.4 software. Quality Assessment of diagnostic Accuracy Version 2 was used to evaluate the risk of bias of each study by Revman 5.3 software. In addition, a meta-regression model was further conducted to explore potential sources of heterogeneity.

**Results:**

According to pre-set inclusion and exclusion criteria, 16 eligible studies were enrolled in this study. A total of 447 CNV eyes and 414 non-CNV eyes were included to investigate the diagnostic accuracy of OCTA. As a result, the pooled sensitivity, specificity, positive likelihood ratio (PLR), negative likelihood ratio (PLR), diagnostic odds ratio (DOR) and the area under the summary receiver operating characteristic curve (sROC-AUC) were 0.87 (95% CI 0.81–0.92), 0.97 (95% CI 0.92–0.99), 32.7 (95% CI 10.1–105.5), 0.13 (95% CI 0.08–0.20), 252 (95% CI 63–1011) and 0.96 (95% CI 0.94–0.97), respectively.

**Conclusions:**

In summary, we demonstrated that OCTA was of high diagnostic value for detecting intraocular CNV.

## Background

Optical coherence tomography angiography (OCTA), based on optical coherence tomography (OCT), is an advanced imaging technology which has recently been approved for clinical ophthalmology application [1]. OCTA is considered as a noninvasive, rapid, secure and repeatable method, which is capable of displaying retinal vasculatures in three-dimensional assessment [2]. Without applying intraocular contrast agents, OCTA, a motion contrast imaging modality, depends on two sorts of backscattered light signals to visualize ophthalmic vascular networks in high-resolution. To be specific, one of the two signals is stable and derives from immobile structures such as neurosensory tissue, while the other is variable and dynamic over time generated by constantly moving tissues like erythrocytes. In comparison with stationary areas, erythrocytes can be measured by repeated B-scans. Thus, an image is created by detecting continuous changes of blood flow. This dye-less imaging technology renders confirmed diagnosis of ocular diseases without the restriction generated by contrast agents.

Pathological myopia, central serous chorioretinopathy (CSCR), age-related macular degeneration (AMD) and uveitides are commonly-detected diseases in ophthalmology, which are characterized by the formation of choroidal neovascularization (CNV) [3–6]. In these disorders, neovascularization is commonly formed in the retina under specific stimulation [7, 8]. Neovascularization is highly permeable, in which hemorrhagic or exudation will occur with the progression of diseases. Under these conditions, visual impairment and irreversible blindness might present in the case of further exacerbated pathological changes.

The prevalence of ophthalmological diseases including pathological myopia and AMD is increasing year by year, which might lead to a potential risk of blindness in the population [9–11]. Severe deterioration of visual acuity will occur by degrees if the progression of CNV is not prevented [12]. To reduce adverse consequences, early detection of CNV will be conducive to increase opportunities for timely and proper treatment. In addition, noninvasive imaging technology is also used for long-term monitoring on eyes which are at high risk of developing CNV [13]. In clinical practice, there are many diagnostic tools for ocular diseases including funduscopy, fluorescein angiography (FA), indocyanine green angiography (ICGA), conventional OCT and OCTA. As a new imaging method, OCTA shows advantages in detecting CNV [14]. In spite of the reported diagnostic accuracy of OCTA for CNV, results are inconsistent with no solid data confirming the exact accuracy of this method. To this end, this study was designed to evaluate the overall diagnostic value of OCTA in the detection of CNV by analyzing different studies.

## Methods

### Search strategy and selection criteria

This study was conducted in accordance with Preferred Reporting Items for Systematic Reviews and Meta-analysis (PRISMA) guidelines [15]. The clinical application of OCTA was first reported in 2014, thus, the initial year of publication was confined to 2014. Three electronic databases including PubMed, Web of Science and Cochrane Library were systematically retrieved by using the following key terms: ‘Optical Coherence Tomography Angiography’ or ‘Optical Coherence Tomography Angiogram’ or ‘OCTA’ and ‘Choroidal Neovascularization’ or ‘Choroid Neovascularization’ or ‘CNV’. Two reviewers screened literatures independently and the deadline of this searching was set at June 2019.

The inclusion criteria were as follows: (1) eyes diagnosed as CNV were included regardless of etiologies; (2) all types of CNV were supposed to be recruited; (3) OCTA was the diagnostic method for CNV compared with at least one dye angiography; (4) sufficient data were available to calculate the true positive, false positive, false negative and true negative.

The exclusion criteria were as follows: (1) conference abstracts, editorials, case reports, letters, reviews and experimental studies; (2) full-text of published articles was not written by English.

### Data extraction

Initial data from each eligible literature were extracted independently by two investigators according to pre-determined research needs: year of publication, first author, country, ethnicity, type of study, number of eyes, etiology of CNV, reference standard, type of CNV, type of OCTA device and diagnostic performance (a true positive, false positive, false negative and true negative). In the case of discrepancy, a third reviewer was consulted to achieve consistent conclusions.

### Quality assessment

Quality Assessment of diagnostic Accuracy Version 2 (QUADAS-2) was used to assess quality of each eligible study [16]. Four domains in QUADAS-2 tool included patient selection, index test, reference standard, and flow and timing. Each item of these domains was required to quantitatively evaluate one by one, which was attentively conducted by two independent investigators through full-text reading. Different results were resolved through consensus by a third reviewer.

### Statistical methods

Above all, the evaluation of threshold effect was conducted according to the Spearman correlation coefficient. A *P*-value less than 0.05 possibly indicated a significant threshold effect. A random-effects model was more suitable to analyze small sample studies. Publication bias was investigated by Deeks’ funnel plot qualitatively and assessed by *P*-value quantificationally. Additionally, a *P*-value over 0.05 suggested no significant publication bias. To be specific, the pooled sensitivity, specificity, positive likelihood ratio (PLR), negative likelihood ratio (NLR) and diagnostic odds ratio (DOR) were major diagnostic evaluation indicators. The summary receiver operator characteristic (sROC) curve was accurately drawn so as to calculate the area under the sROC curve (sROC-AUC). Moreover, Fagan’s Nomogram was conducted to analyze the clinical valuation. For a positive diagnosis, the higher post-test probability than the pre-test probability indicated the great value of the specific method for a definite diagnosis. Otherwise, for a negative diagnosis, a lower post-test probability contributed to the identification of healthy individuals compared with the pre-test probability. In order to obtain reliable results, statistical heterogeneity was supposed to be analyzed by evaluating *I*^2^ and *P*-value quantificationally. If the value of *I*^2^ was more than 50% or *P*-value was less than 0.05, there was significant heterogeneity among these studies. Therefore, a meta-regression model was used to analyze sources of heterogeneity by adding covariates. Heterogeneity analysis of sensitivity and specificity was presented separately. Moreover, a joint model was performed to find out sources of overall heterogeneity. A *P*-value less than 0.05 indicated that this covariate might be the source of heterogeneity. Stata 15.1 and Meta-Disc 1.4 were used for statistical analyses in this meta-analysis. Besides, Revman 5.3 software was implemented to assess the quality of each included literature by QUADAS-2 tool.

## Result

### Study selection and study characteristics

**Fig. 1:**
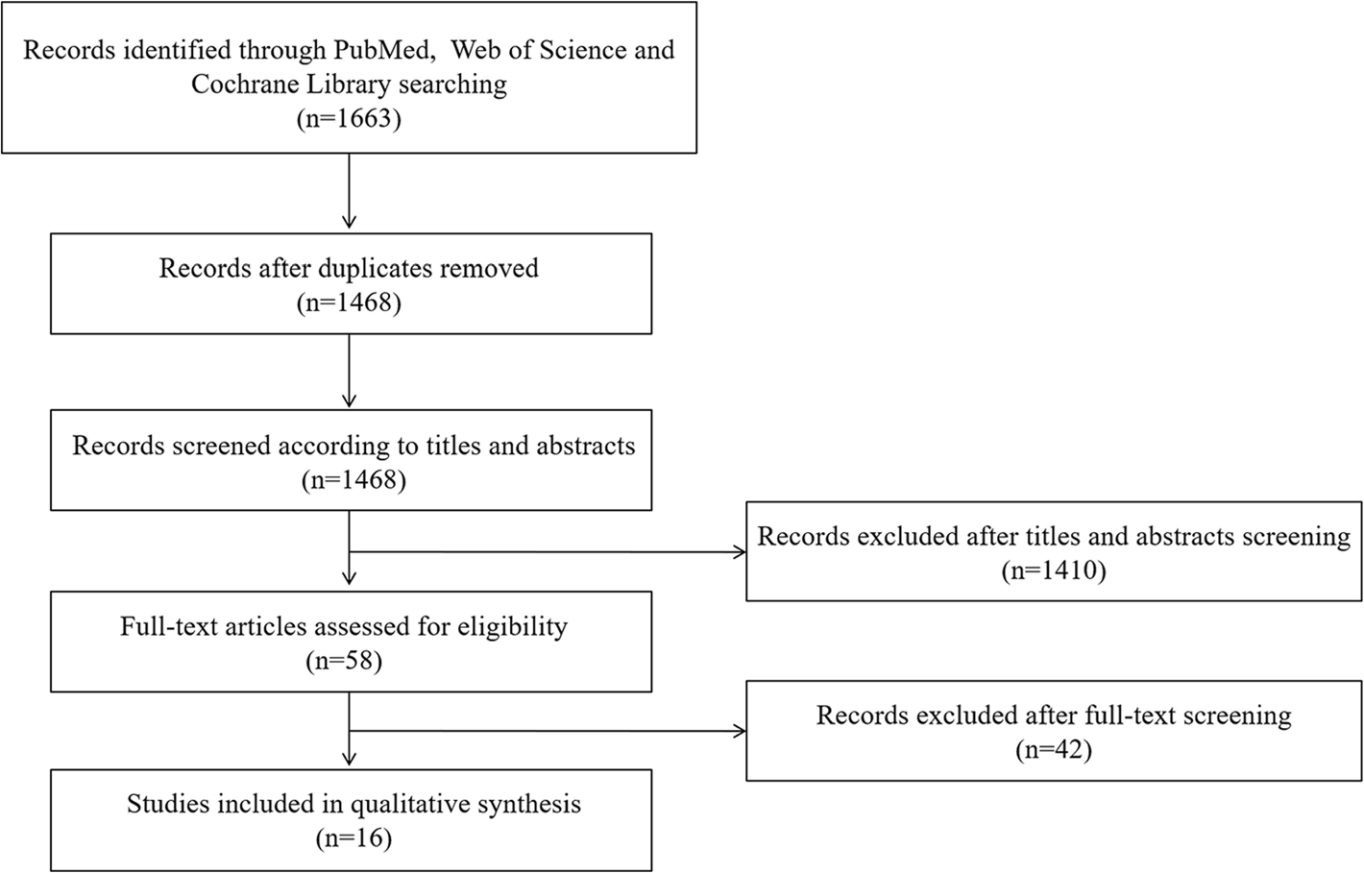
Flow diagram of the selection of studies

As a result, a total of 1663 papers were identified (PubMed: 139, Web of Science: 1473 and Cochrane Library: 51) by using pre-defined search strategy (Fig. 1). According to inclusion and exclusion criteria, 58 full-text articles were finally screened. In total, 16 eligible studies including 447 CNV eyes and 414 non-CNV eyes with sufficient data were selected into the final analysis [17–32]. There were five prospective studies and eight retrospective studies, while the remaining three studies failed to show the type of study. The ethnicity was Asian in two articles while that of other 14 articles was Caucasian or mixed. Sample size of cases ranged from 5 to 107 in each study, which varied from 4 to 63 for controls. In total, the size of eyes sample in eight studies was less than 50, while it reached to more than 50 in the other eight studies. In addition, OCTA was only compared with FA in nine studies, while OCTA was compared with other diagnostic techniques such as ICGA or multimodal imaging in the other seven studies. Device types included AngioVue OCTA (10 studies), Spectralis OCTA (2 studies), AngioPlex OCTA (1 study), Topcon OCTA (1 study). In addition, an earlier study in 2014 used OCTA combining vertical cavity surface emitting laser swept light source OCT with 400 kHz A-scan rate prototype system. Besides, in another study, two types of OCTA devices were utilized, including AngioPlex OCTA and AngioVue OCTA. The etiologies of these CNV were various including AMD, CSCR, adult onset foveomacular vitelliform dystrophy, pathological myopia, reperfused central retinal artery obstruction, adult onset pseudovitelliform lesion, drusenoid pigment epithelium detachment, macroaneurysm, reticular dystrophy, polypoidal choroidal vasculopathy and so on. Of note, all kinds of CNV were included in this study (Table 1).

**Table 1:** Major characteristics of included studies

Author	Year	Tp	Fp	Fn	Tn	Country	Ethnicity	Type of study	Etiology	Reference image	Type of CNV	Device
Moult E	2014	16	0	1	63	USA	Caucasian	Prospective	AMD	FA,ICGA	NA	VCSEL
Lupidi M	2015	4	0	1	20	France	Caucasian	Retrospective	AOFVD	FA,ICGA,OCT	NA	Spectralis
Bonini Filho MA	2015	8	0	0	19	USA	Mixed	Prospective	CSCR	FA	Type1, mixed type 1/2	AngioVue
de Carlo TE	2015	4	2	4	20	USA	Mixed	Retrospective	Multiple1	FA	NA	AngioVue
Shaimov TB	2015	33	1	4	14	Russia	Caucasian	Prospective	AMD	FA	Type1,2	AngioVue
Carnevali A	2016	18	0	4	22	Italy	Caucasian	NA	AMD	ICGA	NA	AngioPlex/ Angiovue
Miyata M	2016	16	0	1	4	Japan	Asian	NA	Myopia	FA	Type 2	AngioVue
de Carlo TE	2016	6	1	1	22	USA	Caucasian	Retrospective	CSCR	FA	NA	AngioVue
Gong J	2016	45	11	7	23	China	Asian	Retrospective	AMD	FA	Type1,2,mixed type1/2	AngioVue
Querques L	2017	19	2	2	30	Italy	Caucasian	Retrospective	Myopia	FA,OCT	NA	AngioPlex
Faridi A	2017	32	0	0	40	USA	Caucasian	Prospective	AMD	FA,OCT	NA	AngioVue
Ahmed D	2018	81	0	26	49	Austria	Caucasian	Retrospective	AMD	FA	Type1,2,3,mixed type	Topcon
Nikolopoulou E	2018	44	2	6	18	Italy	Caucasian	Prospective	AMD	FA	Type1,2,3,mixed type1/2	AngioVue
Souedan V	2018	8	1	1	6	France	Caucasian	Retrospective	Multiple2	FA,OCT	All types	AngioVue
Soomro T	2018	32	6	13	26	England	Caucasian	Retrospective	Multiple3	FA	Type1,2	Spectralis
de Oliveira T	2019	9	0	1	12	Brazil	Mixed	NA	Multiple4	FA,ICGA,OCT	NA	AngioVue

*Tn* True positive, *Fp* False positive, *Fn* False negative, *Tn* True negative, *AMD* Age-related macular degeneration, *AOFVD* Adult onset foveomacular vitelliform dystrophy, *CSCR* Central serous chorioretinopathy, *FA* Fluorescein angiography, *ICGA* Indocyanine green angiography, *OCT* Optical coherence tomography, *CNV* Choroidal neovascularization

Multiple1 = AMD, CSCR and different diagnosis (angioid streaks, multifocal choroiditis, myopic degeneration, pars planitis, or an unclear diagnosis with the differential diagnosis including CSCR, neo-vascular AMD, or polypoidal choroidal vasculopathy (PCV)

Multiple2 = AMD, reperfused central retinal artery obstruction, one adult onset pseudovitelliform lesion, drusenoid pigment epithelium detachment, CSCR, macroaneurysm and reticular dystrophy

Multiple3 = AMD, PCV, CSCR or pathological myopia

Multiple4 = AMD, PCV and CSCR

VCSEL = OCTA combining vertical cavity surface emitting laser (VCSEL) swept light source OCT with 400 kHz A-scan rate prototype system

Topcon = Topcon OCT-A (Topcon Corporation, Tokyo, Japan)

AngioVue = AngioVue OCT-A (Optovue, Inc., Freemont, CA)

AngioPlex = AngioPlex OCT-A (Carl Zeiss Meditec, Inc., Dublin, USA)

Spectralis = Spectralis OCT-A (Heidelberg Engineering, Heidelberg, Germany)

NA = Not available

### Quality assessment and publication bias

**Fig. 2:**
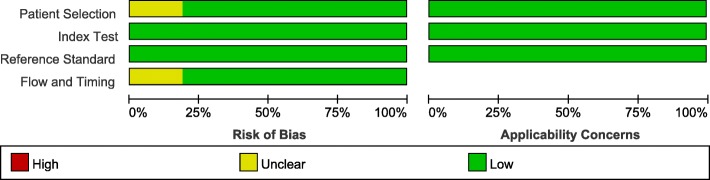
Methodological quality graph by QUADAS-2 of 16 articles

The quality assessment of each study was performed by using QUADAS-2 (shown in Fig. 2), indicating that quality of these studies were almost at moderate to high levels. In order to evaluate the publication bias, Deeks’ funnel plot asymmetry test was conducted. Consequently, a *P*-value of 0.71 suggested no significant publication bias among included studies.

### Diagnostic performance and clinical value

**Fig. 3:**
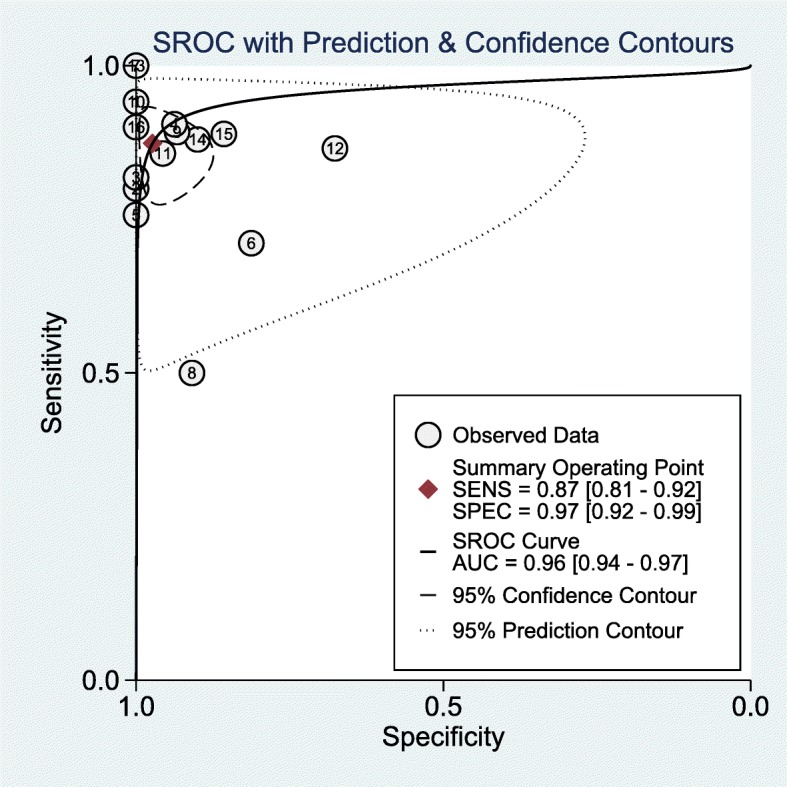
Diagram of sROC curve for assessing the diagnostic value of OCTA in detecting CNV

**Fig. 4:**
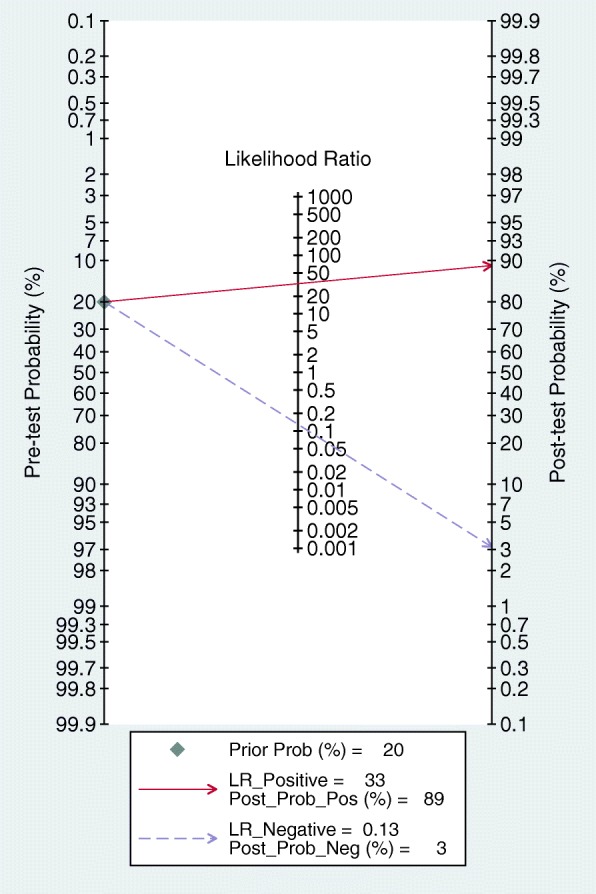
Fagan’s Nomogram of OCTA for CNV

The pooled diagnostic performance of OCTA in detecting CNV showed 0.87 (95% CI 0.81–0.92) of sensitivity, 0.97 (95% CI 0.92–0.99) of specificity and 252 (95% CI 63–1011) of DOR. The sROC curve was showed in Fig. 3, which was 0.96 (95% CI 0.94–0.97). Besides, PLR and NLR were 32.7 (95% CI 10.1–105.5) and 0.13 (95% CI 0.08–0.20), respectively. According to PLR and NLR, the Fagan’s Nomogram was used to obtain 88% of positive post-test probability and 3% of negative post-test probability, when 0.20 of pre-test probability was established in advanced (Fig. 4).

### Heterogeneity analyses

**Fig. 5:**
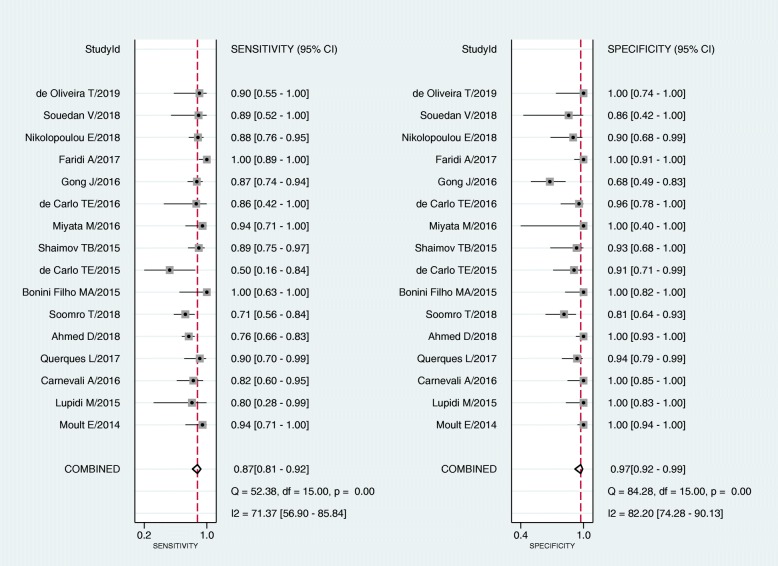
Forest plots of sensitivity and specificity of OCTA in diagnosing CNV

Moreover, the Spearman correlation coefficient was − 0.166 and *P*-value was 0.540, thus, no significant threshold effect existed in this analysis. The *I*^2^ value of 86% with *P*-value < 0.05 suggested significant overall heterogeneity. The forest plots of sensitivity and specificity was conducted, revealing that the *I*^2^ of sensitivity and specificity were 71.37 and 82.20%, respectively (Fig. 5). Therefore, a meta-regression was conducted to perform sources of heterogeneity for analysis of the non-threshold effect. As a result, ethnicity (only Asian / other races), type of study (prospective / retrospective), etiology of CNV (only AMD / other ocular diseases), device of OCTA (only AngioVue / other devices), sample size (≥ 50 / < 50) were not potential sources of heterogeneity in sensitivity (*P*-values ≥0.05). However, *P*-value of reference standard (only FA / other reference standards) was less than 0.01 indicating that it might be a significant source of heterogeneity in sensitivity. The sensitivity of only FA standard group was lower than that of other standards group (0.83 versus 0.92, *p* <  0.01). In terms of specificity, sources of heterogeneity were not found in this study. The results were shown in Table 2.

**Table 2:** Meta-regression and subgroup analyses for sensitivity and specificity

Covariate	Number of studies	Number of eyes	Sensitivity (95% CI)	*P*-value	Specificity (95% CI)	*P*-value
Ethnicity						
Asian	2	107	0.91 (0.80–1.00)	0.69	0.82 (0.53–1.00)	0.09
Other races	14	754	0.87 (0.80–0.93)		0.97 (0.95–1.00)	
Type of study						
Prospective	5	301	0.93 (0.88–0.98)	0.61	0.99 (0.96–1.00)	0.08
Retrospective	8	473	0.79 (0.72–0.86)		0.93 (0.87–1.00)	
Etiology						
AMD	7	560	0.89 (0.82–0.95)	0.12	0.98 (0.94–1.00)	0.44
Other ocular diseases	9	301	0.85 (0.76–0.94)		0.97 (0.92–1.00)	
Reference standard						
FA	9	549	0.83 (0.76–0.90)	< 0.01	0.94 (0.88–1.00)	0.09
Other reference standards	7	372	0.92 (0.86–0.97)		0.99 (0.97–1.00)	
Type of device						
AngioVue	10	426	0.90 (0.86–0.95)	0.09	0.96 (0.91–1.00)	0.66
other devices	6	435	0.80 (0.72–0.87)		0.98 (0.96–1.00)	
Sample size						
≥ 50	8	690	0.88 (0.81–0.95)	0.12	0.96 (0.91–1.00)	0.57
< 50	8	171	0.86 (0.77–0.96)		0.99 (0.96–1.00)	

To explore sources of overall heterogeneity, the joint model of meta-regression was established. Consequently, types of both study and device were potential sources of overall heterogeneity (*p* <  0.01 and *p* = 0.04, respectively), while ethnicity, etiology, reference standard and sample size were not significant sources of overall heterogeneity (*p* = 0.10, *p* = 0.80, *p* = 0.06 and *p* = 0.49, respectively).

## Discussion

Due to the advantages of OCTA in detecting microvascular pathology at various depths, previous studies had focused on the exploration of abnormal vascularity in the eyes by using OCTA images, however, these diagnostic performances failed to reach a consistent conclusion. In 2014, Moult E et al first reported the diagnostic value of OCTA in detecting CNV [17], revealing that 16 of 17 CNV eyes in exudative AMD patients were clearly visualized on OCTA images in comparison with 63 eyes from healthy individuals. The sensitivity and specificity of this exploration were 94 and 100%, respectively. Afterwards, De Carlo TE et al reported only four out of eight CNV eyes were clearly detected on OCTA, while all of the eight CNV eyes were definitely diagnosed in the article of Bonini Filho MA et al [19, 20]. Later, in 2016, Carnevali A et al in Italy and Gong J et al in China reported the diagnosis of CNV based on OCTA imaging [22, 25]. The sensitivity and specificity in the study of Carnevali A et al were 81.8 and 100%, respectively, while they were 86.5 and 67.6%, respectively, in the study of Gong J et al. Subsequently, Ahmed D et al, Faridi A et al and Nikolopoulou E et al also investigated the value of OCTA in detecting CNV in AMD [27–29]. As a result, the sensitivity of these three studies ranged from 75.7 to 88% and the specificity varied from 90 to 100%. Herein, our meta-analysis analyzed the pooled efficacy of OCTA in detecting CNV, showing that the sensitivity, specificity and sROC-AUC were 0.87, 0.97 and 0.96, respectively. Therefore, OCTA technology might render rapid and safe exclusion of non-CNV eyes with high specificity for physicians.

In the elderly, AMD seems to be a major cause for visual impairment and the prevalence of AMD increases with age [33]. Due to the undefined cause of AMD, vascular endothelial growth factor (VEGF) inhibitors are widely applied in most treatments of AMD to prevent the growth of CNV [34]. Therefore, a useful method for monitoring the occurrence of CNV in AMD patients contributes to postponing the deterioration of this disease [35]. Currently, dye angiography is still used as a diagnostic golden standard for CNV; however, it is an invasive dye technology, with risks of side effects after injecting intravenous contrast agents [36]. In spite of accurate identification of CNV patients, the invasive injury could not be avoided for suspected patients without CNV. In addition, OCT is also a technology of detecting neovascular AMD, but it is not a single diagnostic tool owing to limitations in sensitivity and specificity [37]. Compared with OCT, OCTA is a more optimal technology and has been widely applied in the clinical recently. At present, AMD appears to be the most common disease, which might develop CNV. Therefore, in this study, seven articles were collected and analyzed, including 560 eyes that reported the diagnostic value of OCTA in only AMD related CNV. The sensitivity and specificity of OCTA to AMD related CNV were not significantly different from those of other ocular diseases (*p* = 0.12 and *p* = 0.44). In AMD patients, the occurrence of CNV in one eye may lead to CNV risk in the other eye. In a randomized clinical trial, 727 patients with CNV in one single eye were enrolled to receive a ranibizumab or bevacizumab therapy for the other eye. Two years after the treatment, 19% patients developed CNV in the other eye [38]. Thus, if OCTA technology could be performed to monitor CNV in the other eye, 88% of post-test probability in CNV positive eyes and 3% of post-test probability in CNV negative eyes would be reached. Together, these findings demonstrated that OCTA was a reliable imaging technology in early detection of CNV in AMD patients who had developed a CNV eye, which was also able to exclude individuals without CNV. For suspicious CNV eyes, it is unrealistic to receive constant dye angiography for monitoring disease progression, rather, it is promising to use OCTA as a surveillance and screening method.

In general, types of both study and device were potential sources of overall heterogeneity in this meta-analysis. The diagnostic value between the five prospective studies including 305 eyes was significantly different from that in the eight prospective studies including 473 eyes (*p* < 0.01). Compared with prospective studies, retrospective studies might underestimate diagnostic value due to the possible biases caused by inaccurate information recall by researchers. Moreover, to some extent, different kinds of devices might lead to imaging differences in diagnosis. As we have known, the projection artifact is a common problem affecting the diagnostic accuracy of the image equipment in detecting diseases. One of the reasons for projection artifacts is associated with the imaging equipment itself. The difference between AngioVue device group including 10 studies (426 eyes) is significantly different from other devices group including six studies (435 eyes) (*P*-value was 0.04). The sensitivity and specificity of AngioVue group were 0.90 and 0.96, respectively, while they were 0.83 and 0.98, respectively in other devices group. In addition, the reference standard was a source of heterogeneity in sensitivity between only FA group and other reference standards group (*p* < 0.01). The sensitivity in seven other reference standards studies including 312 eyes was higher than nine FA studies including 549 eyes (0.92 versus 0.83). Of note, FA is a common reference standard of the diagnosis of ocular vascular diseases due to the advantage of showing the leakage of neovascularization. The principle of OCTA is different from FA, for it is a quantitative equipment and provides neovascular network information. Souedan V et al used the multimodal imaging as the diagnostic reference standard to assess the diagnostic value of OCTA for CNV [30]. As a result, they found that OCTA showed a higher diagnostic value than FA, for the sensitivity and specificity of OCTA were 85.62 and 81.51%, respectively, and the sensitivity and specificity of FA for CNV were 74.5 and 82.35%, respectively. Besides, compared with FA, other clinical diagnostic methods such as ICGA and OCT also have their own advantages. Other reference standards including multimodal imaging may contribute to the correct classification of diseases, which may lead to a higher sensitivity in other reference standards group in our study.

Additionally, the difference between Asian people and other racial people was also analyzed in the present study. Differences in sensitivity, specificity and overall diagnostic value of ethnicity were not statistically significant, for *P*-values were 0.69, 0.09 and 0.10, respectively. In consideration of the influence of sample size, 16 articles were divided into two subgroups based on whether the sample size of each study was less than 50. As a result, there was no significant difference, thus, sample size was not the source of heterogeneity.

There were certain limitations in our meta-analysis based on published articles. To begin with, in this study, we included all types of CNV to analyze the overall diagnostic value for all kinds of CNV. The diagnostic value of OCTA for different types of CNV might be different, however, the information of each study was insufficient to analyze the difference. Thus, we failed to analyze whether the type of CNV was the source of heterogeneity. Secondly, the sample size of several studies was less than 50 and some studies were retrospective ones in our meta-analysis. To this end, more prospective studies with large sample are warranted to confirm the diagnostic ability of OCTA for CNV in the future. Thirdly, only two studies reported the diagnostic value of OCTA only in Asian population, therefore, more studies from different countries are expected in the future.

In conclusion, OCTA is a potential and reliable method for detecting CNV in ocular diseases based on the sensitivity, specificity and AUC. It might be conducive to monitor the other eye in the case of CNV presence in one eye. In addition, more longitudinal studies of large sample are urgently needed to confirm this conclusion.

## Data Availability

All data in this study was available, for they were all collected from published articles through electronic databases.

## Abbreviations

AMD: Age-related macular degeneration
CNV: Choroidal neovascularization
CSCR: Central serous chorioretinopathy
DOR: Diagnostic odds ratio
FA: Fluorescein angiography
ICGA: Indocyanine green angiography
NLR: Negative likelihood ratio
OCT: Optical coherence tomography
OCTA: Optical coherence tomography angiography
PLR: Positive likelihood ratio
PRISMA: Preferred Reporting Items for Systematic Reviews and Meta-analysis
QUADAS-2: Quality Assessment of diagnostic Accuracy Version 2
SROC: The summary receiver operating characteristic
SROC-AUC: The area under the sROC curve
